# Early pregnancy serum IGFBP-1 relates to lipid profile in overweight and obese women

**DOI:** 10.1016/j.heliyon.2020.e04788

**Published:** 2020-08-30

**Authors:** Kati Mokkala, Juuso Juhila, Noora Houttu, Timo Sorsa, Kirsi Laitinen

**Affiliations:** a Institute of Biomedicine, Research Centre for Integrative Physiology and Pharmacology, University of Turku, Turku, Finland; b Actim Oy, Espoo, Finland; c Department of Oral and Maxillofacial Disease, University of Helsinki and Helsinki University Hospital, Helsinki, Finland; d Department of Oral Diseases, Karolinska Institutet, Huddinge, Sweden; e Department of Obstetrics and Gynecology, Turku University Hospital, Turku, Finland

**Keywords:** Women's health, Pregnancy, Metabolism, Metabolic disorder, Nutrition, Obstetrics & gynecology, IGFBP-1, Insulin, Lipids, MMP-8

## Abstract

Lower level of insulin-like growth factor-binding protein (IGFBP-1) has been observed in insulin resistance, while higher level of matrix metalloproteinase-8 (MMP-8) has been linked to obesity. The aim here was to study in overweight and obese women, typically manifesting with insulin resistance, whether IGFBP-1 and MMP-8 are related to and reflect systemic low-grade inflammation, metabolism and diet. Fasting serum from overweight and obese pregnant women (n = 100) in early pregnancy were analysed for IGFBP-1, phosphorylated IGFBP-1 (phIGFBP-1) and MMP-8. High-sensitivity CRP and GlycA were used as markers for low grade inflammation. GlycA and lipids were quantified using NMR. IGFBP-1 associated negatively with GlycA, evidenced by higher concentrations in the lowest quartile (median 1.53 (IQR 1.45–1.72)) compared to the highest (1.46 (1.39–1.55)) (P = 0.03). Several lipid metabolites, particularly HDL-cholesterol, correlated inversely with phIGFBP-1 (FDR<0.1). Nutritional status and diet contributed to the levels of IGFBP-1, demonstrated as an inverse correlation with maternal weight (Spearman r = -0.205, P = 0.04) and dietary intake of vitamin A (r = -0.253, P = 0.014) and a direct correlation with dietary intake of polyunsaturated fatty acids (Spearman r = 0.222, P = 0.03). MMP-8 correlated inversely with pyridoxine (r = -0.321, P = 0.002) and potassium (r = -0.220, P = 0.033). Maternal serum IGFBP-1 may contribute to maternal lipid metabolism in overweight and obese women during early pregnancy. These findings may be of importance in identification of metabolic disturbances preceding the adverse metabolic outcomes in pregnancy.

## 1 Introduction

Insulin-like growth factor binding protein 1 (IGFBP-1) regulates the half-life and activity of IGF-I and II, with multiple actions on host metabolism and cell growth [1]. Although IGFBP-1 produced by the liver binds only to a small fraction of circulating IGF-1, it is of importance as it’s hepatic production is suppressed by food intake, and subsequently increased glucose and insulin concentrations. On the contrary, at a fasting condition, the production of IGFBP-1 is increased [2]. In addition to systemic effects, both IGF-1 and IGFBP-1 exert profound local roles during pregnancy due to their involvement in implantation and fetal growth. Elevated IGFBP-1 concentrations have been detected in preeclampsia, with both decidua and liver likely contributing to its production [3]. Elevated concentration of decidual phosphorylated IGFBP-1 (phIGFBP-1) has been used as a risk marker for preterm birth [4] and amniotic fluid non-phosphorylated IGFBP-1 as a marker for fetal membrane rupture [5]. Further markers include amniotic fluid inflammatory marker matrix metalloproteinase-8 (MMP-8), the higher concentration of which has been associated with preterm birth [6, 7].

In addition to their crucial roles in pregnancy, elevated serum MMP-8 concentrations have been linked with obesity [8] and with an increased risk for cardiovascular disesases [9], while a lower level of IGFBP-1 has been associated with obesity [10] and with an increased risk for disturbances in glucose metabolism [11]. The roles of MMP-8 and IGFBP-1 as metabolic regulators in pregnancy are less well known, but an inverse association has been observed between serum IGFBP-1 concentrations and the maternal body weight or BMI [12, 13, 14]; there are also reports of an inverse correlation between serum levels of IGFBP-1 and insulin or insulin resistance both in normal weight [15] and in obese [16] pregnant women.

The role of serum MMP-8 with regard to maternal metabolism still awaits clarification. Previous studies in pregnant women have focused on cases of premature birth; elevated levels of MMP-8 were detected in early or mid-trimester amniotic fluid [17]. It is not clear whether circulating levels of IGFBP-1 or MMP-8 are involved in and reflect the inflammatory status of the mother. They may also be linked with maternal glucose and lipid metabolism and thus function as potential regulators of maternal health during pregnancy. In addition, the extent to which IGFBP-1 and MMP-8 may be modified by dietary intake has not been studied, although a link with human nutritional status, like obesity, has been reported.

The aim of this study was to investigate whether serum IGFBP-1 in its non-phosphorylated and phosphorylated forms as well as MMP-8 would associate with serum markers of low-grade inflammation and the metabolic profile in pregnant women at high risk for metabolic disorders, i.e. in overweight and obese women. A secondary aim was to evaluate the relationships of IGFBP-1 and MMP-8 with the degree of obesity as well as the dietary intake of energy yielding nutrients, vitamins and minerals.

## 2 Patients and methods

### 2.1 Study settings

This is a sub-study of a larger mother-infant dietary intervention trial (ClinicalTrials.gov, NCT01922791) being conducted in Southwest Finland and the recruitment initiated at October 2013. The study population consisted of the first 100 overweight pregnant women who provided serum samples at baseline (the first study visit took place at less than 18 wks of pregnancy). Leaflets containing information of the study were distributed in maternal welfare clinics. In addition, media and social media were used to inform about the study. Women interested in participating in the study contacted the project coordinator for further information and to schedule their first study visit, during which their e.g. weight, BMI and body fat percentage were measured.

The inclusion criteria for the intervention trial were prepregnancy BMI≥25, age >18 years, and early pregnancy (max. 18 weeks of gestation). Gestational diabetes in current pregnancy, multifetal pregnancy, and the presence of metabolic or inflammatory disease including type 1 and type 2 diabetes, celiac disease and inflammatory bowel disease were exclusion criteria. In this cross-sectional study, we measured the serum concentrations of IGFBP-1 (phosphorylated and non-phosphorylated form) and MMP-8, which were then related to already measured markers of inflammation (high sensitive C-reactive protein, hsCRP and Glycoprotein acetylation, GlycA), glucose (fasting glucose and insulin, Homeostatic model assessment, (HOMA2-IR) and lipid metabolism (lipid metabolic profile in metabolomics). The study was approved by the Ethics Committee of the Hospital District of Southwest Finland (permission number 115/180/2012). Written informed consent was obtained from all subjects.

### 2.2 Blood sampling and analytical methods of biochemical variables

A fasting (at least 10 h) blood sample was drawn from the antecubital vein of the women on the morning of the study visit (mean 12.8 (SD 2.5) weeks of gestation). The serum was separated and analyzed for hsCRP, insulin and glucose, and the rest of the samples were frozen in aliquots at -80 °C until further analyses.

### 2.3 IGFBP-1 and phIGFBP-1

Concentrations of serum IGFBP-1 and phIGFBP-1 were measured by two immunoenzymometric assays using monoclonal antibodies (Medix Biochemica, Espoo, Finland). The IGFBP-1 assay employing monoclonal antibody 6305 detects the non-phosphorylated and the less phosphorylated isoforms of IGFBP-1, while the phIGFBP-1 assay employing monoclonal antibody 6303 recognizes the highly phosphorylated forms [18]. The detection limit of both assays was 0.3 ng/ml [19]. The data is expressed as ng/ml.

### 2.4 MMP-8

MMP-8 was quantified with a solid-phase immunoenzymometric assay (MMP-8 IEMA, Medix Biochemica, Espoo, Finland [6, 7, 20]. This sandwich assay uses two monoclonal antibodies against human MMP-8. Microplate wells are coated with one monoclonal antibody against MMP-8. The other antibody is conjugated to HRP forming the enzyme conjugate used to detect the presence of MMP-8. Analyses were performed according to the manufacturer's instructions, and the absorbance of the solutions in the wells was measured at 414 nm using a microplate reader (Multiskan, Thermo Fisher Scientific, Vantaa, Finland). The detection limit was 0.04 ng/ml [21]. The data is expressed as ng/ml.

### 2.5 Inflammatory and metabolic markers

The hsCRP was analysed using an automated colorimetric immunoassay on a Dade Behring Dimension RXL autoanalyzer (Siemens Healthcare, Camberly, Surrey, UK), glucose was assayed by an enzymatic method utilizing hexokinase (Cobas 8000 automatic c702-analyzer, Roche Diagnostics GmbH, Mannheim, Germany), and insulin was determined with an immunoelectrochemiluminometric assay (a modular E170 automatic analyzer, Roche Diagnostics GMbH, Mannheim, Germany) in an accredited Turku University Hospital Laboratory which has a quality control system. HOMA2-IR was calculated from fasting plasma glucose and fasting insulin using a HOMA calculator (http://www.dtu.ox.ac.uk/) [22]. GlycA and lipids were quantified from serum samples using a commercial high-throughput proton NMR, metabolomics platform (Nightingale Health Ltd., Helsinki, Finland) as previously described [23]. GlycA is a composite nuclear magnetic resonance (NMR) biomarker of systemic inflammation and consists of N-acetyl sugar groups originating from multiple acute phase circulating glycoproteins: α1-acid glycoprotein, haptoglobin, α1-antitrypsin, α1-antichymotrypsin and transferrin.

### 2.6 Body weight, body mass index and body composition

Prepregnancy BMI (kg/m2) was calculated by dividing self-reported weight in kilograms, obtained from welfare women clinic records, by height measured with a wall stadiometer to the nearest 0.1 cm at the study visit. The weight was measured using an electronic scale and body fat percentage was measured using air displacement plethysmography (Bod Pod system, COSMED, Inc., Concord, CA, USA), at the study visit.

### 2.7 Dietary intake

Three day food diaries were recorded in a week before the study visit. The subjects were advised on how to record their food intake and the diaries were checked for completeness and accuracy with the help of a portion picture booklet. Mean daily dietary intake of energy and nutrients were calculated using computerized software, Aivo diet 2.0.2.3 (Aivo, Turku, Finland), which uses the Food and Nutrient Database of the National Institute for Health and Welfare (Fineli 2016).

### 2.8 Statistics

The normality of the variables was evaluated by performing the Kolmogorov-Smirnov normality test and by visual inspection of the histograms. Since not all of the variables were normally distributed, non-parametric analyses were used, except multiple linear regression which was conducted with natural-logarithmic transformed variables. We used the lowest (Q1) and highest (Q4) quartiles of IGFBP-1, phIGFBP-1 and MMP-8 in the evaluation of the association with the markers of glucose metabolism and inflammation (Mann Whitney u test). In addition, Spearman correlation analyses were performed for comparing serum levels of IGFBP-1, phIGFBP-1 and MMP-8 with serum metabolic markers, inflammation, diet, prepregnancy BMI, weight and body fat percentage. The Benjamini–Hochberg (BH) method was used to control for the false discovery rate of multiple comparisons for the lipid metabolites. The false discovery rate, FDR<0.1 was considered as a statistically significant finding. Multiple linear regression analysis with fasting insulin and prepregnancy BMI were conducted for HDL-particles remaining statistically significant after BH-correction. The results are shown as median (interquartile range, IQR) or as mean (standard deviation, SD) for the parametric characteristics of the women. Statistical analyses were performed with SPSS for Windows, version 23.0 (IBMCorp., Armonk, NY, USA).

## 3 Results

Characteristics of the women are shown in Table 1. The women were studied at mean 12.8 (SD 2.5) weeks of gestation, they were overweight (52%; BMI 25–30 kg/m^2^) or obese (48%; BMI>30 kg/m^2^), the body fat percentage being 44.5% (39.5–48.5%) (median, IQR). Every other woman was highly educated with a college or university degree and 44 % were primipara.

**Table 1:** Characteristics of the women and concentrations of serum biochemical variables (IGFBP-1, phIGFBP-1, MMP-8, hsCRP and GlycA concentrations) in early pregnancy.

Baseline characteristics, n = 100	
Age (years)∗	29 (5)
Highly educated with college or university degree	50%
prepregnancy BMI (kg/m^2^)	29.8 (26.9–32.8)
gestational weeks at sampling∗	12.8 (2.5)
weight (kg) at sampling	83.1 (76.2–92.3)
Smoking before pregnancy (%)	20/92 (21.7%)
Smoking during pregnancy (%)	4/88 (4.3%)

**Metabolic markers**	

IGFBP-1 (ng/ml)	47.0 (26.3–76.8)
phIGFBP-1 (ng/ml)	638.5 (384.8–905.8)
MMP-8 (ng/ml)	19.0 (12.3–29.8)
hsCRP (mg/l)	4.9 (3.0–8.3)
GlycA (mmol/l)	1.5 (1.4–1.6)
Glucose (mmol/l)	4.8 (4.6–5.0)
Insulin mU/L	10.0 (7.3–13.8)
HOMA2-IR	1.3 (0.9–1.7)

∗ mean (SD), others, % or median (IQR).

The values for the biochemical variables are shown in Table 1. The concentration of IGFBP-1 correlated inversely with maternal weight at study visit (Spearman r = -0.205, P = 0.04), whereas neither phIGFBP-1 nor MMP-8 displayed any association with weight (P > 0.239). However, no correlations were observed between prepregnancy BMI (P > 0.063, for all) or body fat percentage (P > 0.085 for all) and IGFBP-1, phIGFBP-1 or MMP-8 levels. In addition, no difference was detected between overweight and obese subjects with respect to their levels of IGFBP-1 (50.0 (30.0–85.0) vs 38.0 (21.0–74.8) ng/ml, respectively, P = 0.093), phIGFBP-1 (660.0 (397.0–964.0) vs 618.0 (308.5–875.3) ng/ml, P = 0.254) or MMP-8 (18.0 (12.0–26.0) vs 20.0 (13.0–32.0) ng/ml, P = 0.495).

### 3.1 Association of IGFBP-1 and phIGFBP-1 with markers of lipid metabolism

**Figure 1:**
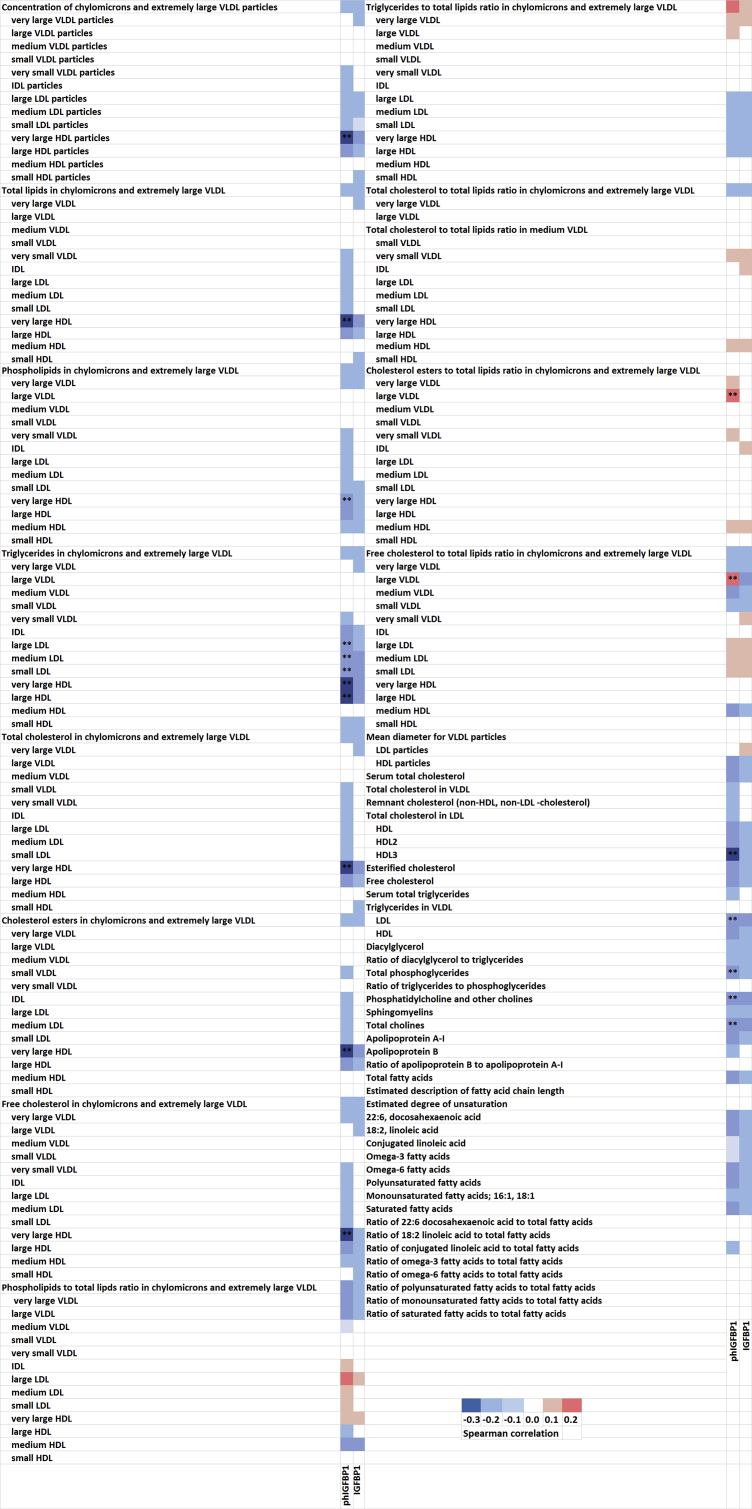
Spearman's correlations between the levels of IGFBP-1 and phIGFBP-1 and serum lipidomic variables. ∗∗ FDR<0.1.

Levels of phIGFBP-1 were related to several lipidomics variables, particularly to HDL-particles (Figure 1a and b, Supplemental Table 1). Interestingly, the strongest correlations were observed between phIGFBP-1 and the lipids in the larger sized particles, such as very large HDL. When correcting for multiple testing (FDR<0.1), 18 correlations remained statistically significant, half of these being (9/18) inverse correlations between phIGFBP-1 and HDL-particles. In contrast, non-phosphorylated IGFBP-1 correlated with very few lipid variables, and these were no longer statistically significant after correction for multiple testing. When investigating the relationship between phIGFBP-1 and HDL-related particles, the associations remained statistically significant even after adjusting with serum insulin and prepregnancy BMI, the correlation being higher between phIGFBP-1 and several HDL-related particles when compared to the that of insulin (Table 2). No correlations were found between MMP-8 and any of the lipid variables (data not shown).

**Table 2:** Association of phIGFBP-1 and insulin with HDL –particles in a linear regression model.

	β (95% CI)	R^2^/P^1^	P-value
Very large HDL concentration (mol/l)			

Insulin (mU/L)	-9.5 × 10^−9^ (-1.8 × 10^−8^ to -1.4 × 10^−9^)	0.181/<0.001	0.022
phIGFBP-1 (ng/ml)	-1.7 × 10^−10^ (-2.7 × 10^−10^ to -6.1 × 10^−11^)		0.002
prepregnancy BMI (kg/m^2^)	-6.8 × 10^9^ (-1.8 × 10^−8^ to 4.3 × 10^−9^)		0.229

Very large HDL-total lipids (mmol/l)			

Insulin (mU/L)	-0.010 (-0.018 to -0.001)	0.181/<0.001	0.002
phIGFBP-1 (ng/ml)	-1.7 × 10^4^ (-2.8 × 10^−4^ to -6.2 × 10^−5^)		0.002
prepregnancy BMI (kg/m^2^)	-0.007 (-0.018 to 0.004)		0.225

Very large HDL-phospolipids (mmol/l)			

Insulin (mU/L)	-0.005 (-0.009 to -0.001)	0.185/<0.001	0.016
phIGFBP-1 (ng/ml)	-8.0 × 10 ^−5^ (-1.3 × 10^−4^ to -2.5 × 10^−5^)		0.004
prepregnancy BMI (kg/m^2^)	-0.004 (-0.009 to 0.002)		0.200

Very large HDL- total cholesterol (mmol/l)			

Insulin (mU/L)	-0.004 (-0.008 to -0.001)	0.181/<0.001	0.026
phIGFBP-1 (ng/ml)	-8.5 × 10^−5^ (-1.4 × 10^−4^ to -3.4 × 10^−5^)		0.001
prepregnancy BMI (kg/m^2^)	-0.003 (-0.008 to 0.002)		0.247

Very large HDL- cholesterol esters (mmol/l)			

Insulin (mU/L)	-0.003 (-0.006 to -4.9 × 10^−4^)	0.183/<0.001	0.022
phIGFBP-1 (ng/ml)	-6.1 × 10^−5^ (- 9.8 × 10^−5^ to -2.5 × 10^−5^)		0.001
prepregnancy BMI	-0.002 (-0.006 to 0.002)		0.279

Very large HDL- free cholesterol (mmol/l)			

Insulin (mU/L)	-0.001 (-0.002 to -1.4 × 10^−5^)	0.169/<0.001	0.047
phIGFBP-1 (ng/ml)	-2. 3 × 10^−5^ (-3.8 × 10^−5^ to -8.0 × 10^−6^)		0.003
prepregnancy BMI (kg/m^2^)	-0.001 (-0.003 to 0.001)		0.181

Very large HDL- triglycerides (mmol/l)			

Insulin (mU/L)	-8.7 × 10^−5^ (-4.4 × 10^−4^ to 3.0 × 10^−4^)	0.069/0.075	0.628
phIGFBP-1 (ng/ml)	-6.1 × 10^−6^ (-1.1 × 10^−5^ to -1.0 × 10^−6^)		0.011
prepregnancy BMI (kg/m^2^)	-1.2 × 10^−4^ (-0.001 to 3.7 × 10^−4^)		0.638

Large HDL- triglycerides (mmol/l)			

Insulin (mU/L)	-4.6 × 10^−4^ (-0.001 to 2.3 × 10^−4^)	0.099/0.018	0.186
phIGFBP-1 (ng/ml)	-2.2 × 10^−5^ (-2.2 × 10^−5^ to -4.0 × 10^−6^)		0.005
prepregnancy BMI (kg/m^2^)	-2.5 × 10^−4^ (-0.001 to 0.001)		0.600

HDL3-cholesterol			

Insulin (mU/L)	-0.001 (-0.003 to 0.001)	0.122/0.006	0.250
phIGFBP-1 (ng/ml)	-4.6 × 10^−5^ (-6.6 × 10^−5^ to -1.4 × 10^5^)		0.003
prepregnancy BMI (kg/m^2^)	-0.002 (-0.004 to 0.001)		0.252

Values are natural log-transformed for linear regression analysis ie. the regression coefficient (β) represent the one-unit change in natural log-scaled phIGFBP-1 (ng/ml), insulin (mU/L) or prepregnancy BMI kg/m^2^ associated with the change of respective HDL-particle in the natural-log-scale. R^2^ = R-square, P^1^ -value = P-value for adjusted linear regression, 95%CI = 95% confidence interval for β.

### 3.2 Association of IGFBP-1, phIGFBP-1 and MMP-8 with inflammatory markers and glucose metabolism

The lower concentration of IGFBP-1 (lowest quartile) was related to a higher concentration of GlycA, a novel marker of low grade inflammation, whereas no differences with the traditional marker of low grade inflammation, hsCRP were observed (Table 3). Furthermore, there were no differences detected in GlycA or hsCRP in relation to phIGFBP-1 or MMP-8 when comparing the lowest and highest quartiles.

**Table 3:** Differences in markers of glucose metabolism and inflammation among IGFBP-1, phlGFBP-1 and MMP-8 quartiles.

IGFBP-1, median ng/ml (range)	Q1, n = 25 15.0 (2.5–26.3)	Q2,n = 28 37.5 (27.0-47.0)	Q3,n = 22 62.5 (48.0–76.0)	Q4,n = 25 101.0 (77.0–147.0)	P Kruskall-Wallis	P Mann-Whitney (Q1-Q4 comparison)
Low grade inflammation						

hsCRP mg/L, all	6.1 (3.8–12.7)	4.3 (2.6–6.6)	4.8 (2.8–8.2)	4.3 (2.1–6.8)	0.166	0.051
GlycA mmol/L	1.53 (1.45–1.72)	1.43 (1.33–1.58)	1.50 (1.42–1.62)	1.46 (1.39–1.55)	0.060	0.030

Glucose metabolism						

Insulin mU/L	13.0 (8.5–17.0)	10.5 (8.0–12.8)	10.0 (6.8–15.0)	9.0 (7–11.5)	0.066	0.007
Glucose mmol/L	4.8 (4.6–5.0)	4.7 (4.5–5.0)	4.8 (2.8–8.2)	4.7 (4.6–5.0)	0.845	0.576
HOMA2IR	1.7 (1.1–2.2)	1.4 (1.1–1.6)	1.2 (0.9–1.9)	1.2 (0.9–1.5)	0.080	0.009

phIGFBP-1, median	Q1, n = 25	Q2, n = 25	Q3, n = 25	Q4,n = 25	P	P

ng/ml (range)	192.0 (57.0–383.0)	522.2 (390.0–636.0)	769.0 (641.0–902.0)	1140.0 (907.0–1860.0)	Kruskall-Wallis	Mann-Whitney (Q1-Q4 comparison)

Low grade inflammation						

hsCRP mg/L	4.9 (3.1–10.0)	4.7 (2.3–9.4)	4.5 (3.1–6.7)	5.1 (3.1–8.2)	0.757	0.756
GlycA mmol/L	1.50 (1.40–1.69)	1.45 (1.32–1.62)	1.53 (1.42–1.60)	1.45 (1.39–1.50)	0.258	0.091

Glucose metabolism						

Insulin mU/L	13.0 (7.2–14.5)	9.0 (7.5–12.0)	10.0 (8.5–13.5)	9.0 (6.0–12.5)	0.191	0.044
Glucose mmol/L	4.8 (4.6–5.0)	4.7 (4.6–4.9)	4.8 (4.6–5.2)	4.7 (4.6–5.0)	0.565	0.899
HOMA2IR	1.6 (1.0–1.9)	1.2 (1.0–1.5)	1.3 (1.1–1.7)	1.2 (0.8–1.6)	0.186	0.047

MMP-8, median	Q1, n = 25	Q2, n = 26	Q3, n = 24	Q4, n = 25	P	P

ng/ml (range)	9.1 (5.0–12.0)	16.3 (13.0–19.0)	23.3 (20.0–29.0)	39.0 (30.0–64.0)	Kruskall-Wallis	Mann-Whitney (Q1-Q4 comparison)

Low grade inflammation						

hsCRP mg/L	4.3 (2.7–8.3)	5.4 (2.8–9.3)	4.4 (2.5–6.3)	5.3 (3.6–8.8)	0.604	0.357
GlycA mmol/L	1.46 (1.35–1.58)	1.48 (1.42–1.62)	1.45 (1.34–1.52)	1.53 (1.44–1.71)	0.168	0.132

Glucose metabolism						

Insulin mU/L	10.0 (7.5–13.0)	10.0 (8.8–13.3)	8.0 (7.0–14.3)	11.0 (8.0–14.5)	0.404	0.284
Glucose mmol/L	4.8 (4.6–5.1)	4.7 (4.6–5.0)	4.8 (4.6–5.0)	4.8 (4.6–5.0)	0.990	0.815
HOMA2IR	1.3 (0.9–1.7)	1.3 (1.1–1.7)	1.0 (0.9–1.8)	1.4 (1.0–1.9)	0.458	0.347

**Figure 2:**
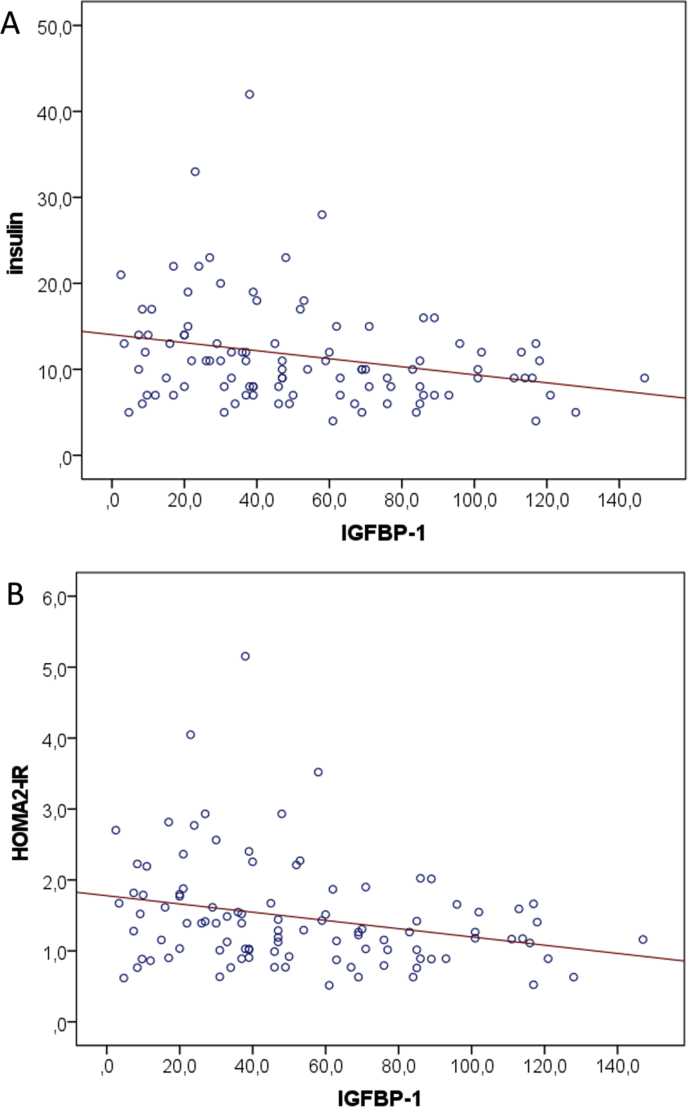
a: Spearman's correlation between concentrations of IGFBP-1 and insulin; b: Spearman's correlation between concentrations of IGFBP-1 and HOMA2-IR.

A lower concentration of IGFBP-1 (lowest quartile) was related to higher levels of both insulin and HOMA2-IR. The same association was detected for the phosphorylated form of IGFBP-1 (Table 3). Further, a statistically significant negative correlation was observed between IGFBP-1 and insulin (Spearman r = -0.270, P = 0.007) (Figure 2a) and HOMA2-IR (r = -0.260, P = 0.009) (Figure 2b). No differences in the variables reflecting glucose metabolism were detected between the highest and lowest quartiles of MMP-8, nor when comparing all the quartiles of IGFBP, phIGFBP or MMP-8. Further, no correlations were detected between IGFBP-1 and glucose or between the levels of phIGFBP-1 or MMP-8 with those of glucose, insulin or HOMA2-IR (data not shown).

### 3.3 Association of dietary intakes of nutrients with IGFBP-1, phIGFBP-1 and MMP-8

A weak, but nonetheless statistically significant positive correlation was observed between the IGFBP-1 concentration and the intake of polyunsaturated fatty acids (PUFA) as the proportion of energy intake (r = 0.222, P = 0.03), while no other correlations were detected between levels of IGFBP-1, or phIGFBP-1 and MMP-8 with the intakes of energy, dietary fibre or energy yielding nutrients; protein, carbohydrates, fat, PUFA, saturated or monounsaturated fatty acids as a proportion of energy intake (P > 0.080, for all). With respect to the dietary intakes of vitamins and minerals, the concentration of retinol correlated inversely with the levels of phIGFBP-1 (r = -0.297, P = 0.004) and IGFBP-1 (r = -0.253, P = 0.014), and there were also inverse correlations between the concentration of MMP-8 with those of pyridoxine (r = -0.321, P = 0.002) and potassium (r = -0.220, P = 0.033).

## 4 Discussion

We revealed a weak negative relation between IGFBP-1 and systemic low-grade inflammation as measured by the novel inflammatory marker GlycA, but not with more traditional marker, hsCRP. In addition to the expected relation between IGFBP-1 and glucose metabolism, we demonstrated using modern metabolomics approach, an inverse correlation between the level of phIGFBP-1 and several lipid variables, particularly large HDL-particles, in overweight and obese women in early pregnancy. The level of MMP- 8 displayed no association with any of the markers of inflammation or glucose and lipid metabolism. Based on these findings, we propose that serum IGFBP-1 reflects maternal metabolism, particularly in relation to lipids during early pregnancy. These findings may be important for maternal health in long-term considering the several metabolic alterations that take place in pregnancy and are further compromised by excess [24].

One new finding emerging from our study utilizing a modern metabolomics approach is that there was an inverse correlation between the level of phIGFBP-1 and several HDL-related particles, which were independent of the serum level of insulin and prepregnancy BMI. In previous studies in non-pregnant study groups, the level of IGFBP-1 has been found to correlate with traditionally measured serum total HDL cholesterol in ischaemic heart disease, type 2 diabetic patients and healthy subjects [25, 26, 27] and in patients with unstable angina [28]. The inverse relationship between phIGFBP-1 and HDL–particles in our study may be related to the physiological alterations in lipid metabolism associated with pregnancy: typically an increase in the amounts of lipids, particularly in triglycerides and total cholesterol has been reported over the course of pregnancy [29]. Furthermore, increasing phosphorylation of IGFBP-1 increases the affinity of IGFBP-1 to IGF-1. Thus, the altered phosphorylation, as manifested by the lower levels of phIGFBP-1, may result in an increased availability of IGF-1, which may beneficially induce the formation of HDL-particles. Another explanation may be related to the activity of lecithin–cholesterol acyltransferase (LCAT) and cholesteryl ester transfer protein (CETP). LCAT is responsible for the transfer of fatty acids to cholesterol and thus for the conversion of cholesterol into cholesterol ester while CETP facilitates the transfer of cholesterol esters from HDL to LDL and VLDL [30]. In this way, IGFBP-1 may participate in the catabolism of large HDL-particles by regulating the synthesis of these proteins. Differing from the traditional analysis for HDL assessment applied in previous studies, we used metabolomics, which provides more detailed information about the HDL-particles, including the particle size and content. Larger HDL-particles have been linked to healthier metabolic outcomes; thus a lower serum phIGFBP-1 concentration may be beneficial with regard to lipid metabolism.

Our finding of the relationship of IGFBP-1 with glucose metabolism is in accordance with previous studies in non-pregnant subjects, in whom lower serum IGFBP-1 concentrations have been analysed in impaired glucose tolerance [11, 31]. Furthermore, reduced IGFBP-1 levels have been detected in women with gestational diabetes when compared to those without this disease [16]. Regardless of the gestational diabetes status, in all of the pregnant women studied, a low IGFBP-1 level was related to increased insulin resistance [16], as also detected in our study.

Low-grade inflammation is suggested to underlie metabolic disturbances. Previous studies have proposed that cytokines, e.g. interleukin-1β increase the production of IGFBP-1 [32]. By using a novel inflammatory marker GlycA, we detected a weak, yet statistically significant inverse association between the levels of GlycA and IGFBP-1 but not with phIGFBP-1 or MMP-8. In a recent study of non-pregnant women with a history of gestational diabetes, a weak correlation was observed between MMP-8 and CRP [33], indicating that MMP-8 may be involved in modulating inflammation. The possible explanation for the lack of a clearer correlation in our study may be that our study population was healthy and without the specific complications typically measured by IGFBP-1, phIGFBP-1 and MMP-8 in late pregnancy. Nevertheless, in general, low-grade inflammation is related to metabolism and the finding of a relationship between IGFBP-1 levels and markers of glucose metabolism and lipidomic profile suggests that IGFBP-1 is involved in maternal metabolism during early pregnancy. We also observed an inverse correlation between the IGFBP-1 concentration and maternal weight, which is in line with previous studies in pregnant women [12, 13, 14]. This suggests that IGFBP-1 may act as a mediator between maternal weight and the alterations in glucose metabolism associated with overweight.

In addition to metabolic and inflammatory variables, using the dietary data, we were able to evaluate the influence of specific nutrients and energy intake on IGFBP-1, phIGFBP-1 and MMP-8. Fasting or malnutrition have been shown to increase the synthesis of IGFBP-1 in a non-pregnant population [2]. We observed a weak association between the intake of PUFA and the IGFBP-1 level and between a few vitamins and minerals and the concentrations of phIGFBP-1 and MMP-8. Based on our results, the potential to modify IGFBP-1 and MMP-8 by dietary means may be limited, although the impact of diet as a regulator of inflammatory status [34] and maternal glucose metabolism has been convincingly demonstrated [35].

Our findings may be of importance considering the metabolic health of pregnant women. The prevalence of metabolic complications, such as gestational diabetes during pregnancy, is increasing in association with increasing overweight and obesity in the population. In addition, these metabolic disturbances during pregnancy increase the risk for later complications, such as type 2 diabetes and cardiovascular diseases. The observed relationship between IGFBP-1 and phIGFBP-1 with maternal metabolism suggests that the IGFBP-1 may be involved in the regulation of maternal metabolic health and thus represent a novel target for intervention. However, the causality and the levels resulting in clinical manifestations need to be established, as previously either lower [11, 30] or higher [36] serum IGFBP-1 concentrations have been measured in association with imbalanced glucose tolerance and cardiovascular complications in non-pregnant population.

The strength of this study lies in its well characterized study population. Furthermore, we obtained comprehensive data from metabolomics to investigate the association between the concentrations of IGFBP-1, phIGFBP-1 and MMP-8 with maternal metabolism. We consider one limitation of this study is that all of the analyses were carried during early pregnancy. Further studies will be needed to assess the role of IGFBP-1, phIGFBP-1 and MMP-8 in maternal metabolism throughout the entire course of pregnancy, both in normal weight women and in relation to clinical manifestations like gestational diabetes.

## 5 Conclusions

We found that the level of IGFBP-1, particularly in its phosphorylated form, correlated with the amounts of several lipid particles especially with lipids in large HDL particles. Furthermore, a weak inverse correlation between IGFBP-1 and inflammation was observed. Our findings suggest that the IGF-system, which has an important role in fetal development and in regulation of glucose metabolism, may also participate in maternal lipid metabolism during early pregnancy.

## Declarations

### Author contribution statement

K. Laitinen: Conceived and designed the experiments; Analyzed and interpreted the data; Contributed reagents, materials, analysis tools or data; Wrote the paper.

K. Mokkala: Conceived and designed the experiments; Performed the experiments; Analyzed and interpreted the data; Contributed reagents, materials, analysis tools or data; Wrote the paper.

J. Juhila: Conceived and designed the experiments; Performed the experiments; Contributed reagents, materials, analysis tools or data.

N. Houttu and T. Sorsa: Contributed reagents, materials, analysis tools or data.

### Funding statement

K. Laitinen was supported by State research funding for university-level health research of the Turku University Hospital Expert Responsibility Area and Academy of Finland (#258606). N. Houttu was supported by Turku University Foundation. T. Sorsa was supported by Huch (2016252, 2017252, 2018229, Y10114SLO17, Y10114SLO18).

### Competing interest statement

The authors declare the following conflict of interests: T. Sorsa and J. Juhila are inventors of diagnostic patents 127416 and US 2017/0023671A1.

### Additional information

The clinical trial described in this paper was registered at ClinicalTrials.gov under the registration number NCT01922791.
