# Corrigendum to “Early pregnancy serum IGFBP-1 relates to lipid profile in overweight and obese women” [Heliyon 6 (8) (August 2020) Article e04788]

**DOI:** 10.1016/j.heliyon.2020.e05248

**Published:** 2020-10-22

**Authors:** Kati Mokkala, Juuso Juhila, Noora Houttu, Timo Sorsa, Kirsi Laitinen

**Affiliations:** a Institute of Biomedicine, Research Centre for Integrative Physiology and Pharmacology, University of Turku, Turku, Finland; b Actim Oy, Espoo, Finland; c Department of Oral and Maxillofacial Disease, University of Helsinki and Helsinki University Hospital, Helsinki, Finland; d Department of Oral Diseases, Karolinska Institutet, Huddinge, Sweden; e Department of Obstetrics and Gynecology, Turku University Hospital, Turku, Finland

In the original published version of this article, Figure 2A was incorrectly duplicated as a copy of Figure 2B. Figure 2A has now been replaced with the correct figure. The authors apologize for this mistake. Both the HTML and PDF versions of the article have been updated to correct the error.

