# Supplementary material for: Early pregnancy serum IGFBP-1 relates to lipid profile in overweight and obese women
Source: Heliyon. 2020 Aug 30;6(8):e04788. doi: 10.1016/j.heliyon.2020.e04788 (PMC7475181; doi:10.1016/j.heliyon.2020.e04788)
Supplement: Supplemental table 1 [file mmc1.pdf]

Supplemental Table 1. Correlations between the levels of IGFBP-1, phIGFB-1 and lipidomic variables.

	phIGFBP1			IGFBP1		
	Spearman's rho	P-value	Benjamini-Hochberg adjusted P-value	Spearman's rho	P-value	Benjamini-Hochberg adjusted P-value
Concentration of chylomicrons and extremely large VLDL particles (mol/l)	-0,11	0,275	0,469	-0,152	0,131	0,537
Concentration of very large VLDL particles (mol/l)	-0,069	0,498	0,689	-0,111	0,272	0,653
Concentration of large VLDL particles (mol/l)	-0,007	0,944	0,971	-0,037	0,716	0,882
Concentration of medium VLDL particles (mol/l)	-0,002	0,985	0,992	-0,002	0,986	0,994
Concentration of small VLDL particles (mol/l)	-0,066	0,512	0,704	-0,039	0,701	0,872
Concentration of very small VLDL particles (mol/l)	-0,167	0,098	0,289	-0,072	0,479	0,756
Concentration of IDL particles (mol/l)	-,198*	0,048	0,2	-0,097	0,339	0,675
Concentration of large LDL particles (mol/l)	-0,19	0,058	0,229	-0,105	0,299	0,675
Concentration of medium LDL particles (mol/l)	-0,185	0,065	0,244	-0,11	0,276	0,653
Concentration of small LDL particles (mol/l)	-0,183	0,069	0,244	-0,102	0,312	0,675
Concentration of very large HDL particles (mol/l)	-,312**	0,002	0,053	-,216*	0,031	0,537
Concentration of large HDL particles (mol/l)	-,235*	0,019	0,135	-0,165	0,101	0,537
Concentration of medium HDL particles (mol/l)	-0,099	0,327	0,526	-0,095	0,348	0,68
Concentration of small HDL particles (mol/l)	-0,077	0,449	0,642	-0,148	0,141	0,549
Total lipids in chylomicrons and extremely large VLDL (mmol/l)	-0,114	0,26	0,447	-0,155	0,123	0,537
Total lipids in very large VLDL (mmol/l)	-0,072	0,477	0,677	-0,114	0,258	0,639
Total lipids in large VLDL (mmol/l)	-0,011	0,911	0,951	-0,041	0,688	0,862
Total lipids in medium VLDL (mmol/l)	-0,003	0,98	0,992	-0,001	0,991	0,994
Total lipids in small VLDL (mmol/l)	-0,077	0,443	0,642	-0,047	0,645	0,838
Total lipids in very small VLDL (mmol/l)	-0,166	0,099	0,289	-0,068	0,502	0,775
Total lipids in IDL (mmol/l)	-0,179	0,075	0,25	-0,079	0,434	0,739
Total lipids in large LDL (mmol/l)	-0,182	0,07	0,244	-0,096	0,343	0,676
Total lipids in medium LDL (mmol/l)	-0,174	0,083	0,259	-0,098	0,33	0,675
Total lipids in small LDL (mmol/l)	-0,175	0,082	0,259	-0,093	0,356	0,689
Total lipids in very large HDL (mmol/l)	-,310**	0,002	0,053	-,215*	0,032	0,537
Total lipids in large HDL (mmol/l)	-,226*	0,024	0,146	-0,157	0,118	0,537

Total lipids in medium HDL (mmol/l)	-0,096	0,342	0,536	-0,089	0,376	0,691
Total lipids in small HDL (mmol/l)	-0,083	0,412	0,605	-0,154	0,126	0,537
Phospholipids in chylomicrons and extremely large VLDL (mmol/l)	-0,15	0,137	0,348	-0,182	0,07	0,537
Phospholipids in very large VLDL (mmol/l)	-0,121	0,23	0,419	-0,154	0,125	0,537
Phospholipids in large VLDL (mmol/l)	-0,04	0,693	0,829	-0,063	0,533	0,775
Phospholipids in medium VLDL (mmol/l)	-0,019	0,849	0,913	-0,01	0,918	0,971
Phospholipids in small VLDL (mmol/l)	-0,059	0,561	0,731	-0,033	0,746	0,903
Phospholipids in very small VLDL (mmol/l)	-0,179	0,075	0,25	-0,075	0,461	0,755
Phospholipids in IDL (mmol/l)	-0,175	0,082	0,259	-0,073	0,471	0,756
Phospholipids in large LDL (mmol/l)	-0,17	0,09	0,274	-0,088	0,381	0,691
Phospholipids in medium LDL (mmol/l)	-0,17	0,092	0,276	-0,099	0,327	0,675
Phospholipids in small LDL (mmol/l)	-0,194	0,054	0,221	-0,112	0,268	0,653
Phospholipids in very large HDL (mmol/l)	-,272**	0,006	0,091	-0,184	0,066	0,537
Phospholipids in large HDL (mmol/l)	-,234*	0,019	0,135	-0,17	0,092	0,537
Phospholipids in medium HDL (mmol/l)	-0,148	0,141	0,348	-0,136	0,176	0,563
Phospholipids in small HDL (mmol/l)	-0,001	0,995	0,995	-0,092	0,36	0,691
Triglycerides in chylomicrons and extremely large VLDL (mmol/l)	-0,104	0,304	0,514	-0,144	0,153	0,549
Triglycerides in very large VLDL (mmol/l)	-0,06	0,556	0,731	-0,1	0,321	0,675
Triglycerides in large VLDL (mmol/l)	-0,004	0,97	0,992	-0,038	0,704	0,872
Triglycerides in medium VLDL (mmol/l)	0,015	0,881	0,929	0,004	0,965	0,983
Triglycerides in small VLDL (mmol/l)	-0,019	0,854	0,914	0,006	0,953	0,981
Triglycerides in very small VLDL (mmol/l)	-0,136	0,179	0,386	-0,067	0,509	0,775
Triglycerides in IDL (mmol/l)	-,238*	0,017	0,129	-0,135	0,182	0,568
Triglycerides in large LDL (mmol/l)	-,277**	0,005	0,089	-0,195	0,051	0,537
Triglycerides in medium LDL (mmol/l)	-,285**	0,004	0,085	-,217*	0,03	0,537
Triglycerides in small LDL (mmol/l)	-,263**	0,008	0,095	-,209*	0,037	0,537
Triglycerides in very large HDL (mmol/l)	-,317**	0,001	0,053	-,227*	0,023	0,537
Triglycerides in large HDL (mmol/l)	-,315**	0,001	0,053	-,223*	0,026	0,537
Triglycerides in medium HDL (mmol/l)	-0,028	0,781	0,88	-0,042	0,676	0,862

Triglycerides in small HDL (mmol/l)	-0,126	0,21	0,403	-0,147	0,144	0,549
Total cholesterol in chylomicrons and extremely large VLDL (mmol/l)	-0,134	0,182	0,386	-0,172	0,087	0,537
Total cholesterol in very large VLDL (mmol/l)	-0,09	0,375	0,566	-0,14	0,165	0,549
Total cholesterol in large VLDL (mmol/l)	-0,016	0,876	0,928	-0,05	0,62	0,825
Total cholesterol in medium VLDL (mmol/l)	-0,024	0,813	0,902	-0,012	0,906	0,971
Total cholesterol in small VLDL (mmol/l)	-0,125	0,217	0,404	-0,088	0,381	0,691
Total cholesterol in very small VLDL (mmol/l)	-0,101	0,316	0,526	-0,005	0,958	0,981
Total cholesterol in IDL (mmol/l)	-0,132	0,189	0,389	-0,033	0,746	0,903
Total cholesterol in large LDL (mmol/l)	-0,147	0,145	0,351	-0,063	0,533	0,775
Total cholesterol in medium LDL (mmol/l)	-0,148	0,142	0,348	-0,076	0,45	0,755
Total cholesterol in small LDL (mmol/l)	-0,148	0,14	0,348	-0,071	0,482	0,756
Total cholesterol in very large HDL (mmol/l)	-,331**	0,001	0,053	-,227*	0,023	0,537
Total cholesterol in large HDL (mmol/l)	-,200*	0,046	0,2	-0,128	0,203	0,584
Total cholesterol in medium HDL (mmol/l)	-0,043	0,672	0,809	-0,048	0,636	0,832
Total cholesterol in small HDL (mmol/l)	-0,099	0,325	0,526	-0,129	0,202	0,584
Cholesterol esters in chylomicrons and extremely large VLDL (mmol/l)	-0,134	0,185	0,386	-0,174	0,084	0,537
Cholesterol esters in very large VLDL (mmol/l)	-0,035	0,726	0,852	-0,098	0,332	0,675
Cholesterol esters in large VLDL (mmol/l)	0,058	0,568	0,733	0,007	0,948	0,981
Cholesterol esters in medium VLDL (mmol/l)	-0,02	0,844	0,913	-0,01	0,918	0,971
Cholesterol esters in small VLDL (mmol/l)	-0,127	0,208	0,403	-0,091	0,368	0,691
Cholesterol esters in very small VLDL (mmol/l)	-0,077	0,446	0,642	0,007	0,944	0,981
Cholesterol esters in IDL (mmol/l)	-0,117	0,247	0,438	-0,019	0,848	0,961
Cholesterol esters in large LDL (mmol/l)	-0,145	0,149	0,355	-0,065	0,521	0,775
Cholesterol esters in medium LDL (mmol/l)	-0,143	0,157	0,367	-0,071	0,483	0,756
Cholesterol esters in small LDL (mmol/l)	-0,135	0,18	0,386	-0,062	0,54	0,775
Cholesterol esters in very large HDL (mmol/l)	-,339**	0,001	0,053	-,238*	0,017	0,537
Cholesterol esters in large HDL (mmol/l)	-,200*	0,046	0,2	-0,131	0,194	0,584

Cholesterol esters in medium HDL (mmol/l)	-0,02	0,845	0,913	-0,032	0,751	0,904
Cholesterol esters in small HDL (mmol/l)	-0,057	0,576	0,735	-0,076	0,455	0,755
Free cholesterol in chylomicrons and extremely large VLDL (mmol/l)	-0,134	0,184	0,386	-0,175	0,081	0,537
Free cholesterol in very large VLDL (mmol/l)	-0,125	0,214	0,403	-0,163	0,105	0,537
Free cholesterol in large VLDL (mmol/l)	-0,082	0,415	0,605	-0,105	0,299	0,675
Free cholesterol in medium VLDL (mmol/l)	-0,051	0,617	0,764	-0,041	0,686	0,862
Free cholesterol in small VLDL (mmol/l)	-0,098	0,33	0,526	-0,058	0,57	0,778
Free cholesterol in very small VLDL (mmol/l)	-0,139	0,167	0,382	-0,027	0,789	0,936
Free cholesterol in IDL (mmol/l)	-0,157	0,118	0,318	-0,049	0,627	0,83
Free cholesterol in large LDL (mmol/l)	-0,158	0,117	0,318	-0,064	0,526	0,775
Free cholesterol in medium LDL (mmol/l)	-0,162	0,108	0,31	-0,08	0,431	0,739
Free cholesterol in small LDL (mmol/l)	-0,185	0,065	0,244	-0,097	0,335	0,675
Free cholesterol in very large HDL (mmol/l)	-,307**	0,002	0,053	-,199*	0,047	0,537
Free cholesterol in large HDL (mmol/l)	-,203*	0,043	0,2	-0,129	0,2	0,584
Free cholesterol in medium HDL (mmol/l)	-0,129	0,202	0,403	-0,109	0,279	0,653
Free cholesterol in small HDL (mmol/l)	-0,087	0,392	0,583	-0,142	0,159	0,549
Phospholipids to total lipds ratio in chylomicrons and extremely large VLDL (%)	-,233*	0,02	0,137	-0,165	0,102	0,537
Phospholipids to total lipds ratio in very large VLDL (%)	-,216*	0,034	0,177	-0,196	0,054	0,537
Phospholipids to total lipds ratio in large VLDL (%)	-,220*	0,029	0,158	-0,137	0,177	0,563
Phospholipids to total lipds ratio in medium VLDL (%)	-0,118	0,242	0,433	-0,058	0,569	0,778
Phospholipids to total lipds ratio in small VLDL (%)	0,055	0,583	0,739	-0,01	0,921	0,971
Phospholipids to total lipds ratio in very small VLDL (%)	-0,035	0,732	0,852	0,029	0,776	0,929
Phospholipids to total lipds ratio in IDL (%)	0,12	0,232	0,419	-0,001	0,994	0,994

Phospholipids to total lipds ratio in large LDL (%)	,220*	0,028	0,157	0,111	0,273	0,653
Phospholipids to total lipds ratio in medium LDL (%)	0,138	0,171	0,383	0,066	0,515	0,775
Phospholipids to total lipds ratio in small LDL (%)	0,124	0,218	0,404	0,055	0,59	0,795
Phospholipids to total lipds ratio in very large HDL (%)	0,148	0,141	0,348	0,127	0,206	0,585
Phospholipids to total lipds ratio in large HDL (%)	0,126	0,21	0,403	0,017	0,869	0,971
Phospholipids to total lipds ratio in medium HDL (%)	-,227*	0,023	0,146	-,213*	0,034	0,537
Phospholipids to total lipds ratio in small HDL (%)	0,087	0,39	0,583	0,041	0,684	0,862
Triglycerides to total lipids ratio in chylomicrons and extremely large VLDL (%)	,208*	0,038	0,193	0,144	0,153	0,549
Triglycerides to total lipids ratio in very large VLDL (%)	0,164	0,109	0,31	0,143	0,161	0,549
Triglycerides to total lipids ratio in large VLDL (%)	0,133	0,19	0,389	0,082	0,421	0,735
Triglycerides to total lipids ratio in medium VLDL (%)	0,092	0,363	0,552	0,022	0,826	0,961
Triglycerides to total lipids ratio in small VLDL (%)	0,059	0,563	0,731	0,048	0,637	0,832
Triglycerides to total lipids ratio in very small VLDL (%)	-0,069	0,498	0,689	-0,091	0,367	0,691
Triglycerides to total lipids ratio in IDL (%)	-0,06	0,551	0,729	-0,059	0,56	0,778
Triglycerides to total lipids ratio in large LDL (%)	-0,128	0,206	0,403	-0,138	0,172	0,563
Triglycerides to total lipids ratio in medium LDL (%)	-0,177	0,078	0,256	-0,185	0,065	0,537

Triglycerides to total lipids ratio in small LDL (%)	-0,134	0,185	0,386	-0,157	0,119	0,537
Triglycerides to total lipids ratio in very large HDL (%)	-0,14	0,164	0,38	-0,122	0,228	0,615
Triglycerides to total lipids ratio in large HDL (%)	-,199*	0,047	0,2	-0,147	0,145	0,549
Triglycerides to total lipids ratio in medium HDL (%)	0,063	0,531	0,716	0,056	0,58	0,787
Triglycerides to total lipids ratio in small HDL (%)	-0,099	0,328	0,526	-0,089	0,38	0,691
Total cholesterol to total lipids ratio in chylomicrons and extremely large VLDL (%)	-0,157	0,118	0,318	-0,117	0,247	0,639
Total cholesterol to total lipids ratio in very large VLDL (%)	-0,055	0,59	0,744	-0,046	0,656	0,847
Total cholesterol to total lipids ratio in large VLDL (%)	0,038	0,712	0,847	0,021	0,836	0,961
Total cholesterol to total lipids ratio in medium VLDL (%)	-0,097	0,338	0,533	-0,027	0,791	0,936
Total cholesterol to total lipids ratio in small VLDL (%)	-0,057	0,576	0,735	-0,019	0,853	0,961
Total cholesterol to total lipids ratio in very small VLDL (%)	0,114	0,26	0,447	0,115	0,256	0,639
Total cholesterol to total lipids ratio in IDL (%)	0,065	0,523	0,71	0,102	0,31	0,675
Total cholesterol to total lipids ratio in large LDL (%)	0,033	0,747	0,865	0,085	0,402	0,714
Total cholesterol to total lipids ratio in medium LDL (%)	0,014	0,888	0,932	0,059	0,557	0,778
Total cholesterol to total lipids ratio in small LDL (%)	0,026	0,797	0,889	0,074	0,466	0,756

Total cholesterol to total lipids ratio in very large HDL (%)	-0,1	0,323	0,526	-0,088	0,383	0,691
Total cholesterol to total lipids ratio in large HDL (%)	-0,027	0,787	0,882	0,071	0,481	0,756
Total cholesterol to total lipids ratio in medium HDL (%)	0,126	0,213	0,403	0,117	0,247	0,639
Total cholesterol to total lipids ratio in small HDL (%)	-0,021	0,837	0,913	0,003	0,976	0,99
Cholesterol esters to total lipids ratio in chylomicrons and extremely large VLDL (%)	-0,093	0,355	0,548	-0,044	0,662	0,849
Cholesterol esters to total lipids ratio in very large VLDL (%)	0,123	0,231	0,419	0,083	0,42	0,735
Cholesterol esters to total lipids ratio in large VLDL (%)	,268**	0,007	0,093	,247*	0,014	0,537
Cholesterol esters to total lipids ratio in medium VLDL (%)	-0,022	0,83	0,911	0,019	0,85	0,961
Cholesterol esters to total lipids ratio in small VLDL (%)	-0,032	0,755	0,866	-0,01	0,925	0,971
Cholesterol esters to total lipids ratio in very small VLDL (%)	0,114	0,259	0,447	0,081	0,425	0,736
Cholesterol esters to total lipids ratio in IDL (%)	0,098	0,331	0,526	0,124	0,22	0,609
Cholesterol esters to total lipids ratio in large LDL (%)	0,008	0,936	0,968	0,06	0,552	0,778
Cholesterol esters to total lipids ratio in medium LDL (%)	-0,035	0,726	0,852	0,014	0,887	0,971
Cholesterol esters to total lipids ratio in small LDL (%)	-0,03	0,77	0,872	0,025	0,803	0,945
Cholesterol esters to total lipids ratio in very large HDL (%)	-0,06	0,551	0,729	-0,099	0,325	0,675

Cholesterol esters to total lipids ratio in large HDL (%)	0,017	0,867	0,923	0,097	0,338	0,675
Cholesterol esters to total lipids ratio in medium HDL (%)	0,159	0,115	0,318	0,143	0,157	0,549
Cholesterol esters to total lipids ratio in small HDL (%)	-0,002	0,987	0,992	0,021	0,835	0,961
Free cholesterol to total lipids ratio in chylomicrons and extremely large VLDL (%)	-0,174	0,084	0,259	-0,13	0,196	0,584
Free cholesterol to total lipids ratio in very large VLDL (%)	-0,185	0,069	0,244	-0,163	0,111	0,537
Free cholesterol to total lipids ratio in large VLDL (%)	-,288**	0,004	0,085	-,241*	0,016	0,537
Free cholesterol to total lipids ratio in medium VLDL (%)	-,262**	0,009	0,101	-0,178	0,076	0,537
Free cholesterol to total lipids ratio in small VLDL (%)	-0,193	0,055	0,221	-0,122	0,226	0,615
Free cholesterol to total lipids ratio in very small VLDL (%)	0,047	0,645	0,785	0,176	0,08	0,537
Free cholesterol to total lipids ratio in IDL (%)	-0,053	0,602	0,754	0,015	0,883	0,971
Free cholesterol to total lipids ratio in large LDL (%)	0,115	0,254	0,447	0,115	0,253	0,639
Free cholesterol to total lipids ratio in medium LDL (%)	,199*	0,048	0,2	0,14	0,164	0,549
Free cholesterol to total lipids ratio in small LDL (%)	0,149	0,138	0,348	0,101	0,319	0,675
Free cholesterol to total lipids ratio in very large HDL (%)	-0,093	0,359	0,55	0,024	0,815	0,954
Free cholesterol to total lipids ratio in large HDL (%)	-0,094	0,352	0,547	0,006	0,955	0,981

Free cholesterol to total lipids ratio in medium HDL (%)	-,226*	0,024	0,146	-0,153	0,128	0,537
Free cholesterol to total lipids ratio in small HDL (%)	-0,086	0,394	0,583	-0,062	0,542	0,775
Mean diameter for VLDL particles (nm)	0,06	0,55	0,729	-0,016	0,878	0,971
Mean diameter for LDL particles (nm)	0,047	0,642	0,785	0,101	0,316	0,675
Mean diameter for HDL particles (nm)	-,252*	0,011	0,107	-0,168	0,095	0,537
Serum total cholesterol (mmol/l)	-,216*	0,031	0,165	-0,125	0,214	0,6
Total cholesterol in VLDL (mmol/l)	-0,101	0,316	0,526	-0,06	0,556	0,778
Remnant cholesterol (non-HDL, non-LDL -cholesterol) (mmol/l)	-0,148	0,142	0,348	-0,075	0,457	0,755
Total cholesterol in LDL (mmol/l)	-0,145	0,15	0,355	-0,066	0,513	0,775
Total cholesterol in HDL (mmol/l)	-,221*	0,027	0,155	-0,157	0,12	0,537
Total cholesterol in HDL2 (mmol/l)	-,202*	0,044	0,2	-0,14	0,165	0,549
Total cholesterol in HDL3 (mmol/l)	-,302**	0,002	0,053	-,199*	0,048	0,537
Esterified cholesterol (mmol/l)	-,222*	0,026	0,154	-0,134	0,184	0,568
Free cholesterol (mmol/l)	-,228*	0,022	0,146	-0,186	0,064	0,537
Serum total triglycerides (mmol/l)	-0,125	0,214	0,403	-0,099	0,329	0,675
Triglycerides in VLDL (mmol/l)	-0,022	0,829	0,911	-0,019	0,849	0,961
Triglycerides in LDL (mmol/l)	-,274**	0,006	0,091	-,201*	0,045	0,537
Triglycerides in HDL (mmol/l)	-,246*	0,014	0,129	-0,193	0,054	0,537
Diacylglycerol (mmol/l)	-0,134	0,183	0,386	-0,115	0,253	0,639
Ratio of diacylglycerol to triglycerides (mmol/l)	-0,127	0,209	0,403	-0,103	0,309	0,675
Total phosphoglycerides (mmol/l)	-,263**	0,008	0,095	-0,196	0,05	0,537
Ratio of triglycerides to phosphoglycerides	0,071	0,485	0,684	0,062	0,541	0,775
Phosphatidylcholine and other cholines (mmol/l)	-,268**	0,007	0,093	-,203*	0,042	0,537
Sphingomyelins (mmol/l)	-,199*	0,047	0,2	-0,156	0,121	0,537
Total cholines (mmol/l)	-,276**	0,005	0,089	-,213*	0,033	0,537
Apolipoprotein A-I (g/l)	-,244*	0,015	0,129	-0,163	0,105	0,537

Apolipoprotein B (g/l)	-0,154	0,127	0,338	-0,079	0,437	0,739
Ratio of apolipoprotein B to apolipoprotein A-I	-0,002	0,983	0,992	0,063	0,531	0,775
Total fatty acids (mmol/l)	-,241*	0,016	0,129	-0,189	0,059	0,537
Estimated description of fatty acid chain length, not actual carbon number	0,01	0,925	0,961	-0,07	0,488	0,759
Estimated degree of unsaturation	-0,035	0,732	0,852	-0,013	0,9	0,971
22:6, docosahexaenoic acid (mmol/l)	-,201*	0,044	0,2	-0,157	0,118	0,537
18:2, linoleic acid (mmol/l)	-,237*	0,017	0,129	-0,153	0,129	0,537
Conjugated linoleic acid (mmol/l)	-0,182	0,07	0,244	-0,114	0,257	0,639
Omega-3 fatty acids (mmol/l)	-0,182	0,071	0,244	-0,151	0,134	0,539
Omega-6 fatty acids (mmol/l)	-,257**	0,01	0,107	-0,168	0,095	0,537
Polyunsaturated fatty acids (mmol/l)	-,254*	0,011	0,107	-0,175	0,081	0,537
Monounsaturated fatty acids; 16:1, 18:1 (mmol/l)	-0,183	0,069	0,244	-0,156	0,12	0,537
Saturated fatty acids (mmol/l)	-,241*	0,016	0,129	-0,187	0,062	0,537
Ratio of 22:6 docosahexaenoic acid to total fatty acids	-0,047	0,645	0,785	-0,052	0,606	0,812
Ratio of 18:2 linoleic acid to total fatty acids (%)	-0,03	0,77	0,872	0,034	0,734	0,899
Ratio of conjugated linoleic acid to total fatty acids (%)	-0,139	0,169	0,383	-0,085	0,398	0,712
Ratio of omega-3 fatty acids to total fatty acids (%)	0,052	0,607	0,756	0,011	0,911	0,971
Ratio of omega-6 fatty acids to total fatty acids (%)	-0,066	0,516	0,705	0,012	0,909	0,971
Ratio of polyunsaturated fatty acids to total fatty acids (%)	-0,069	0,496	0,689	-0,012	0,908	0,971
Ratio of monounsaturated fatty acids to total fatty acids (%)	0,044	0,663	0,802	0,014	0,892	0,971

Ratio of saturated fatty acids to total fatty acids (%)	-0,031	0,756	0,866	-0,058	0,566	0,778
